# Policy Series: The Impact of Ageism on Health and Efforts to Address It Through Reframing Aging

**DOI:** 10.1093/geroni/igaa057.2556

**Published:** 2020-12-16

**Authors:** Erica Solway

## Abstract

Ageism can manifest in a variety of ways including in institutional policies and practices, interpersonal interactions, older adults’ internalization of ageist stereotypes, and through the media. While major incidents of age-based discrimination and their negative effects on health are more recognized, older adults also experience less momentous, but still harmful ageism in their day-to-day lives. These types of everyday ageism are also harmful to health. This symposium will highlight recent research from the University of Michigan National Poll on Healthy Aging on self-reported experiences of everyday ageism among a nationally representative sample of U.S. adults age50-80 and its connections to physical and mental health outcomes. Next, another researcher also using data from the National Poll on Healthy Aging will explore the prevalence of positive attributes of aging, its relationships to health, and ways in which it may buffer older adults from the adverse health consequences of ageism. Then speakers will highlight the Reframing Aging initiative and the ways in which GSA and other organizations are leading a national effort to address ageism through education and dissemination of research-based resources. This session will feature research, education, and advocacy efforts and will focus on the policy implications of efforts to better understand the prevalence of and strategies to counter the effects of ageism.

